# Dimethyl Fumarate Alleviates Dextran Sulfate Sodium-Induced Colitis, through the Activation of Nrf2-Mediated Antioxidant and Anti-inflammatory Pathways

**DOI:** 10.3390/antiox9040354

**Published:** 2020-04-24

**Authors:** Shiri Li, Chie Takasu, Hien Lau, Lourdes Robles, Kelly Vo, Ted Farzaneh, Nosratola D. Vaziri, Michael J. Stamos, Hirohito Ichii

**Affiliations:** 1 Department of Surgery, University of California, Irvine, CA 92868, USA; shiril@hs.uci.edu (S.L.); takasu.chie@tokushima-u.ac.jp (C.T.); hlau2@uci.edu (H.L.); lyrobles@llu.edu (L.R.); kmvo89@gmail.com (K.V.); mstamos@hs.uci.edu (M.J.S.); 2 Department of Pathology, University of California, Irvine, CA 92868, USA; sfarzane@uci.edu; 3 Department of Medicine, University of California, Irvine, CA 92868, USA; ndvaziri@uci.edu

**Keywords:** colitis, Nrf2, dimethyl fumarate, antioxidant, anti-inflammation

## Abstract

Oxidative stress and chronic inflammation play critical roles in the pathogenesis of ulcerative colitis (UC) and inflammatory bowel diseases (IBD). A previous study has demonstrated that dimethyl fumarate (DMF) protects mice from dextran sulfate sodium (DSS)-induced colitis via its potential antioxidant capacity, and by inhibiting the activation of the NOD-, LRR- and pyrin domain-containing protein 3 (NLRP3) inflammasome. This study aims to clarify the nuclear factor erythroid 2-related factor 2/antioxidant responsive element (Nrf2/ARE) pathway pharmacological activation and anti-inflammatory effect by DMF, through focusing on other crucial antioxidant enzymes and inflammatory mediator, including glutamate-cysteine ligase catalytic subunit (GCLC), glutathione peroxidase (GPX) and cyclooxygenase-2 (COX-2), in a DSS-induced colitis mouse model. The oral administration of DMF attenuated the shortening of colons and alleviated colonic inflammation. Furthermore, the expression of key antioxidant enzymes, including GCLC and GPX, in the colonic tissue were significantly increased by DMF administration. In addition, protein expression of the inflammatory mediator, COX-2, was reduced by DMF administration. Our results suggest that DMF alleviates DSS-induced colonic inflammatory damage, likely via up-regulating GCLC and GPX and down-regulating COX-2 protein expression in colonic tissue.

## 1. Introduction

Ulcerative colitis (UC) is one of the main forms of inflammatory bowel diseases (IBD), characterized by the chronic inflammation of the gastrointestinal tract with a poorly understood mechanism [1]. The pathogenesis of UC has been proposed to involve oxidative stress and elevated mucosal immune response [2]. Various medications are being explored as a treatment for UC, as current anti-inflammatory and immunosuppressive agents are insufficient, due to their serious side effects and ineffectiveness [3]. Dimethyl fumarate (DMF) has been demonstrated to have both anti-inflammatory and antioxidant effects in different inflammatory diseases, with mild side effects [4]. Fumaderm, an oral formulation of DMF combined with fumaric esters, has been used to treat psoriasis for over 15 years [5]. Recently, the Food and Drug Administration and European Medicines Agency has approved BG-12, an oral form of DMF, for the treatment of multiple sclerosis (MS) [6]. While the mechanism of action of DMF is unclear, DMF is hypothesized to exert its effects by activating the kelch-like ECH-associated protein 1–nuclear factor erythroid 2-related factor 2–antioxidant-responsive element (Keap1-Nrf2-ARE) oxidative stress response pathway in the animal model of MS, leading to the enhanced expression of various antioxidant enzymes, such as glutamate-cysteine ligase catalytic subunit (GCLC), NAD(P)H quinone oxidoreductase 1 (NQO1), heme oxygenase-1 (HO-1), glutathione peroxidase (GPX), etc. [7] In the dextran sulfate sodium (DSS)-induced colitis mouse model of IBD, Liu et al. have shown that treatment with DMF induced the activation of the Nrf2-ARE pathway, resulting in the upregulation of its target antioxidant enzymes, including NQO1 and HO-1 [8]. In addition, the anti-inflammatory effects of DMF have been attributed to the Nrf2-dependent inhibition of NOD-, LRR- and pyrin domain-containing protein 3 (NLRP3) inflammasome activation and the suppression of pro-inflammatory mediators, such as interleukin 1 beta (IL-1β), interleukin 6 (IL-6), tumor necrosis factor alpha (TNF- α) and nuclear factor kappa B (NF-κB) [8,9]. 

Our previous study, exploring the effects of DMF on attenuating chronic pancreatitis, has shown that HO-1 expression was significantly upregulated in pancreatic tissue after incubation in DMF [10]. Similarly, we demonstrated that the oral administration of DMF prevented tissue damage in liver ischemia/reperfusion injury by increasing the expression of antioxidant enzymes, including catalase (CAT) and glutamate-cysteine ligase modifier subunit (GCLM), but not GCLC, GPX, HO-1 or NQO-1, suggesting that the mechanism of DMF action could be tissue specific [11]. Moreover, liver tissues treated with DMF had decreased expression levels of inflammation mediators NF-kB and cyclooxygenase-2 (COX-2), and pro-inflammatory cytokines and chemokines, including cluster of differentiation 86 (CD86), IL-6, interleukin 10 (IL-10), TNF-a, etc. [11] This study aimed to clarify the pharmacological effects of DMF on nuclear factor erythroid 2-related factor 2/antioxidant responsive element (Nrf2/ARE) pathway activation and the anti-inflammatory system, by focusing on other crucial antioxidant enzymes and inflammatory mediators, including GCLC, GPX and COX-2 in the DSS-induced colitis mouse model.

## 2. Material and Methods

### 2.1. Animals, Experimental Design, and DMF Administration

**Figure 1:**
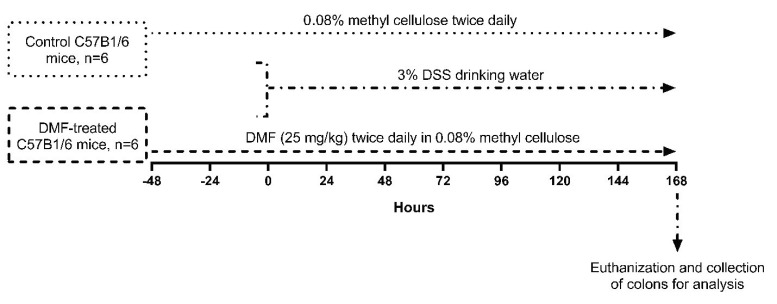
Experimental design outlining the dextran sulfate sodium (DSS)-induced colitis mice model and dimethyl fumarate (DMF) treatment protocol. In the DMF-treated C57B1/6 mice group (*n* = 6), DMF was dissolved in 0.08% methyl cellulose and given to mice (25 mg/kg) twice daily by oral gavage for 48 h prior to the administration of DSS, and maintained throughout the experiment. Control C57B1/6 mice (*n* = 6) were given the same amount of methyl cellulose. Both groups were given 3% DSS drinking water for 1 week to induce intestinal inflammation. All mice were then euthanized and their colons were collected for analysis.

All animal procedures were performed in accordance with the Institutional Animal Care and Use Committee (IACUC #2013-3091, approved on May 12, 2013) at the University of California, Irvine. The 8-week old male C57B1/6 mice were obtained from Charles River company (Wilmington, MA, USA). All animals were maintained under standard pathogen-free conditions (room temperature: 22 °C, humidity: 50 ± 5%, 12:12 h light/dark cycle, and free access to food and water). Stock solutions of DMF (Sigma, MO, USA) were dissolved in 0.08% methyl cellulose (Sigma, MO, USA) and given to mice (25 mg/kg), twice daily by oral gavage. In control group (*n* = 6), the same amount of methyl cellulose was fed as a vehicle by oral gavage [11]. In the treated group (*n* = 6), DMF was given to mice (25 mg/kg) twice daily for 48 h prior to initiating DSS administration, and maintained throughout the experiment. All mice were given 3% dextran sodium sulfate (DSS, molecular weight 36,000-50,000, MP Biochemicals, Santa Ana, CA, USA) through drinking water for 1 week to induce intestinal inflammation. After 1 week, the mice were euthanized and their entire colons were collected for analysis (Figure 1).

### 2.2. Histopathological Analysis

Parts of the colon tissue were fixed in 10% neutral buffered formalin and embedded in paraffin. The fixed tissues were processed into 5 um sections and stained with hematoxylin and eosin. The severity of DSS-induced colitis was blindly graded [12]. Scoring of the histological damage of colon tissues was based on 3 parameters. The severity of inflammation was scored as follows: 0, rare inflammatory cells in the lamina propria; 1, increased numbers of granulocytes in the lamina propria; 2, confluence of inflammatory cells extending into the submucosa; 3, transmural extension of the inflammatory infiltrate. The damage to colon crypts was scored as follows: 0, intact crypts; 1, loss of the basal one-third; 2, loss of the basal two-thirds; 3, entire crypt loss; 4, change of epithelial surface with erosion; 5, confluent erosion. Ulceration was scored as follows: 0, absence of ulcer; 1, 1 or 2 foci of ulcerations; 2, 3 or 4 foci of ulcerations; 3, confluent or extensive ulceration. Values were added to give a maximal histological score of 11.

### 2.3. Protein Extraction and Western Blots Analysis 

Colon tissues were homogenized. The total protein was then extracted using a CelLytic™ NuCLEAR™ Extraction Kit (Sigma), according to the manufacturer’s instruction. The protein concentration was quantified using the Bio-Rad DC Protein Assay Kit (Bio-Rad Laboratories, Hercules, CA, USA). The following primary antibodies were used for Western blot analysis: rabbit antibodies against glutamate-cysteine ligase catalytic subunit (GCLC) (Abcam Inc, Cambridge, MA, USA), glutathione peroxidase (GPX) (Abcam Inc, Cambridge, MA, USA) and cyclooxygenase-2 (COX-2) (Abcam Inc, Cambridge, MA, USA). Then, 50 ug protein aliquots were incubated at 55 °C for 5 min. The heated samples were loaded with NuPAGE 4–12% Bis-Tris gel (Life Technologies, Grand Island, NY, USA) and transferred to a polyvinylidene difluoride membrane (Pall Life Sciences, Ann Arbor, MI, USA). After blocking in 5% blocking grade non-fat dry milk TBS-T (Thermo Fisher Scientific, Waltham, MA, USA), the membrane was incubated overnight at 4 °C with primary antibodies. Following a wash, the sample was incubated with a HRP-conjugated goat anti-rabbit secondary antibody for 2 h at RT. A chemiluminescence imaging system (Thermo Fisher Scientific, Waltham, MA, USA) was used to image immunoreactive bands. ImageQuant (Molecular Dynamics, Caesarea, Israel) was used to perform densitometric measurements. The expression of target proteins was normalized to the GAPDH housekeeping protein, then the normalized intensities were divided by the intensity of the control group and expressed as relative protein level to their controls.

### 2.4. Statistical Analysis

All results were presented as mean ± SEM. An unpaired student’s *t*-test was used to evaluate the significance between control and DMF-treated groups. Statistical significance was defined as a *p*-value less than 0.05.

## 3. Results

### 3.1. DMF Treatment Mitigated DSS-Induced Murine Colitis

**Figure 2:**
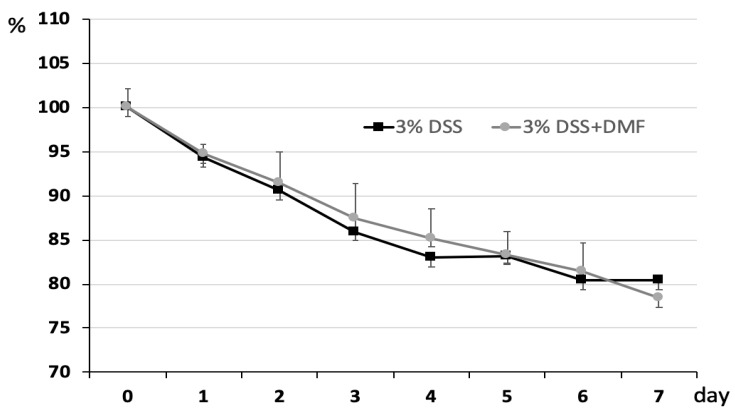
Body weight change. Mice were weighed daily to assess body weight loss. Each point represents the mean ± SEM.

**Figure 3:**
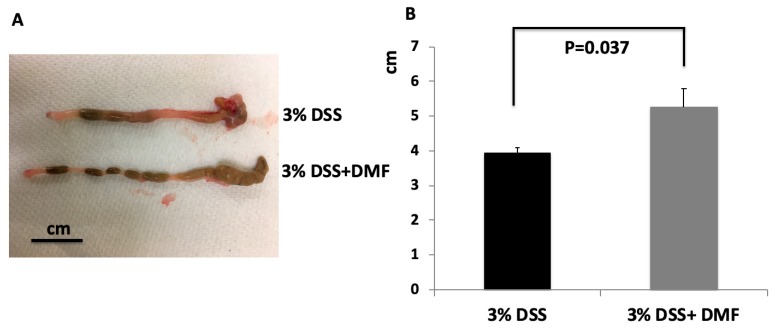
(**A**) Photograph of representative colon at day 7 of DSS administration. (**B**) Length of colons from mice given 3% dextran sulfate sodium (DSS) and 3% DSS+ dimethyl fumarate (DMF) (25 mg/kg twice daily). Data represent the mean ± SEM.

**Figure 4:**
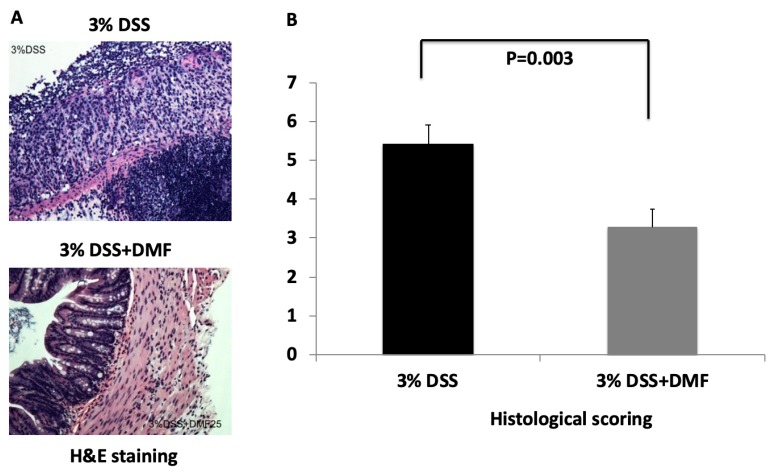
Histopathological examination of colon tissues from mice given 3% dextran sulfate sodium (DSS) and 3% DSS+ dimethyl fumarate (DMF) (25 mg/kg, twice daily). (**A**) Hematoxylin and eosin (H&E)-stained histology of colon tissues from DSS-induced colitis mice, with and without DMF treatment, magnification: ×200. Histopathological analysis of colon tissues of mice without DMF treatment showed a substantial loss of epithelium and increase in the infiltration of inflammatory cells, whereas colon tissues from mice treated with DMF had a marked reduction in inflammatory cell infiltration. (**B**) Histology damage scores of colon tissues from DSS-induced colitis mice, with and without DMF treatment. Data represent the mean ± SEM.

The severity of DSS-induced colitis was monitored by measuring daily change of body weight. As shown in Figure 2, loss of body weight was observed in both groups. There were no significant differences at any time points between the two groups. To explore the protective effect of DMF on colon injury after induction of UC, the length of the entire colon was measured, and colon tissue was stained with H + E for histological analysis. Mice treated with DMF had significantly reduced colonic shortening compared to untreated control mice (DMF = 5.25 ± 0.53 cm vs. control = 3.92 ± 0.18 cm; *p* = 0.037) (Figure 3B). Histopathological analysis revealed the loss of epithelium and increased infiltration of inflammatory cells in the untreated control mice. (Figure 4A). In contrast, colon tissues from mice treated with DMF showed reduced inflammatory cell infiltration and significantly lower histopathological score (DMF = 3.27 ± 0.46 vs. control = 5.42 ± 0.50 cm; *p* = 0.003) (Figure 4A,B), indicating that DMF treatment ameliorated the UC-induced histological changes. 

### 3.2. Effects of DMF Treatment on Protein Expression of Antioxidant Enzymes in Colon Tissue

**Figure 5:**
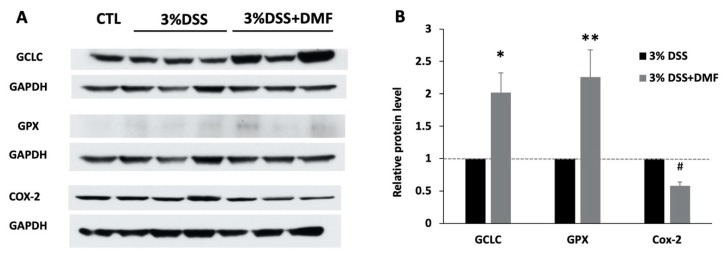
Western blot of colon tissues from mice given 3% dextran sulfate sodium (DSS) and 3% DSS+ dimethyl fumarate (DMF) (25 mg/kg twice daily). (**A**) Representative western blots of glutamate-cysteine ligase catalytic subunit (GCLC), glutathione peroxidase (GPX), and cyclooxygenase-2 (COX-2) from colon tissues of DSS-induced colitis mice, with and without DMF treatment. (**B**) Relative protein level to control group. GCLC (*n* = 5), GPX (*n* = 5), COX-2 (*n* = 6), data represent the mean ± SEM. * *p* = 0.028 or ** *p* = 0.04 or # *p* = 0.001 versus control group.

A similar study has reported that DMF causes the translocation of Nrf2 to the nucleus, leading to the upregulation of its antioxidant enzymes [8]. Western blots analysis showed that the protein expression level of GCLC in colon tissues was 2.02-fold greater in DMF-treated group than in control group, and the GPX level was 2.26-folder greater than in the control group (Figure 5).

### 3.3. Effects of DMF Treatment on Protein Expression of COX-2

Colonic tissues from DMF-treated mice had significantly reduced COX-2 protein expression compared to placebo-treated mice, by as much as 42% (Figure 5). 

## 4. Discussion

Ulcerative colitis (UC) is a chronic inflammatory disease of the gastrointestinal tracts that is reportedly due to an increased infiltration of inflammatory cells and upregulation of pro-oxidant molecules. In this study, we evaluated the effects of DMF on a mouse model of DSS-induced colitis. Our results showed that DMF treatment ameliorated DSS-induced colitis in mice, confirming the result of a previous study by Liu et al [8]. 

Previous works have reported that oxidative stress has a critical role in the pathogenesis of UC [2]. Our previous study has shown that DMF exerts antioxidant effects by upregulating the protein expressions of CAT, GCLM, and endothelial nitric oxide synthase (eNOS), but not NQO1, HO-1, GCLC or GPX in the liver tissue of mice with liver reperfusion injury [11]. Moreover, in addition to activating NQO1 and HO-1, studies in MS have found that DMF increases the expression of GCLC and GPX [13], which indicates that the antioxidant pathway activated by DMF may be tissue-specific. 

In this study, we have specifically verified that targeted antioxidant genes of the Nrf2 pathway, GCLC and GPX, were upregulated after DMF treatment in mice with DSS-induced colitis. GCLC is a subunit of the enzyme GCL that regulates the synthesis of glutathione (GSH) [14]. GSH is a tripeptide thiol that functions to detoxify electrophiles and scavenge reactive oxygen and nitrogen species [15]. A reduced level of GSH has been reported in both the animal model of colitis and patients with UC [16,17]. The treatment of GSH precursor, *N*-acetylcysteine, has been shown to raise GSH level and improve mucosal function in experimental colitis [18,19]. GPX has a major role in the GSH antioxidant pathway by reducing hydrogen, lipid, and organic peroxides, while oxidizing GSH into glutathione disulfide (GSSG) [15]. The upregulation of GCLC and GPX by DMF treatment could potentially contribute to the observed decrease in UC-induced damage. 

UC has been shown to be largely due to chronic inflammation with the substantial infiltration of immune cells accompanied by the heightened production of pro-inflammatory mediators, such as NF-κB, TNF-α and COX-2 [20,21,22]. COX-2 expression has also been found to be elevated in experimental colitis [23]. Pro-inflammatory cytokines, such as TNF-α, IL-1β, and IL-6, could upregulate COX-2 expression during an inflammatory response [24]. COX-2 has a major role in the inflammation process, by catalyzing the conversion of arachidonic acid into prostaglandins [25]. DMF treatment before the induction of liver ischemic/reperfusion injury has been found to reduce production of the inflammatory mediators, including TNF-α, COX-2, IL-6 and NF-κB [11]. In a study by Liu et al., oral administration of DMF led to a substantial decrease in the level of TNF-α, IL-1β, and IL-6 in the colon tissues of mice with DSS-induced colitis [8]. Similarly, DMF diminished the expression of NF-κB p65, IL-1β, and TNF-α in experimental colitis [9]. Consistent with these findings, we observed that the treatment of DMF led to the downregulation of COX-2 expression in colonic tissue, which confirmed the anti-inflammatory effect of DMF in mice with UC. 

It is well known that the dysbiosis of gut microbiome plays a key role in the initiation and maintenance of UC [26]. Moreover, there is growing evidence that sulphate-reducing bacteria, an anaerobic microorganism that belongs to normal microbiota in the gastrointestinal tract, can be a trigger of intestinal inflammation and contribute to UC [27,28,29]. Interestingly, some recent studies have shown that treatment of DMF in the patients with MS also has an impact on altering microbiota composition [30,31]. In our current study, we didn’t examine the gut microbiome change. A future study is needed to explore the possible effect of DMF on altering colonic microbiome diversity which may contribute the inhibition of colonic inflammation.

## 5. Conclusions

In conclusion, our study suggests that DMF alleviates DSS-induced colonic inflammatory damage, likely via up-regulating GCLC and GPX and down-regulating COX-2 protein expression in colonic tissue.

## Abbreviations

Ulcerative colitis (UC), inflammatory bowel diseases (IBD), dimethyl fumarate (DMF), multiple sclerosis (MS), kelch-like ECH-associated protein 1 (Keap1), nuclear factor erythroid 2-related factor 2 (Nrf2), antioxidant-responsive element (ARE), heme oxygenase-1 (HO-1), NAD(P)H:quinone oxidoreductase 1 (NQO1), glutamate-cysteine ligase catalytic subunit (GCLC), glutathione peroxidase (GPX), dextran sulfate sodium (DSS), catalase (CAT), glutamate-cysteine ligase modifier subunit (GCLM), cyclooxygenase-2 (COX-2), endothelial nitric oxide synthase (eNOS).
